# Supplementary material for: Panton–Valentine leucocidin is the key determinant of Staphylococcus aureus pyomyositis in a bacterial GWAS
Source: eLife. 2019 Feb 22;8:e42486. doi: 10.7554/eLife.42486 (PMC6457891; doi:10.7554/eLife.42486)
Supplement: Supplementary file 1. [file elife-42486-supp1.docx]

|  | Pyomyositis (2007-2012) | Nasal carriage (2008) | Nasal carriage (2012) |
| --- | --- | --- | --- |
| Number of isolates | 101 | 222 | 195 |
| Age (med, IQR) | 7.8 years  (4.2-11.8) | 5.9 years  (2.5- 8.3) | 6.3 years  (4.1- 9.9) |
| Male (n (%) | 66/97 (68%) | 122/221 (55.2%) | 105/195 (53.8%) |
| MRSA (n (%)) | 0 (0%) | 61 (27.5%) | 52 (26.7%) |

**Supplementary File 1**
