# Supplementary material for: Panton–Valentine leucocidin is the key determinant of Staphylococcus aureus pyomyositis in a bacterial GWAS
Source: eLife. 2019 Feb 22;8:e42486. doi: 10.7554/eLife.42486 (PMC6457891; doi:10.7554/eLife.42486)
Supplement: Transparent reporting form [file elife-42486-transrepform.pdf]

eLIFE
1st Floor
24 Hills Road
Cambridge CB2 1JP, UK
P 01223 855340
W elifesciences.org
T @elife

eLife's transparent reporting form

We encourage authors to provide detailed information *within their submission* to facilitate the interpretation and replication of experiments. Authors can upload supporting documentation to indicate the use of appropriate reporting guidelines for health-related research (see EQUATOR Network), life science research (see the BioSharing Information Resource), or the ARRIVE guidelines for reporting work involving animal research. Where applicable, authors should refer to any relevant reporting standards documents in this form.

If you have any questions, please consult our Journal Policies and/or contact us: editorial@elifesciences.org.

Sample-size estimation

- You should state whether an appropriate sample size was computed when the study was being designed
- You should state the statistical method of sample size computation and any required assumptions
- If no explicit power analysis was used, you should describe how you decided what sample (replicate) size (number) to use

Please outline where this information can be found within the submission (e.g., sections or figure legends), or explain why this information doesn't apply to your submission:

The collection of samples from cases was retrospective over a five year period in a diagnostic microbiology laboratory based at the Angkor Hospital for Children in Siem Reap, Cambodia. All samples collected were used in the study, no power calculation was performed on the number of cases we would include given the retrospective nature of the case samples in the study.
Two sets of control samples were collected from children attending outpatient clinics at the Angkor Hospital for Children, the first carriage study was performed for another study (Nickerson, Wuthiekanun et al. 2011) but was also at the beginning of the retrospective study on pyomyositis, in 2008 where 2,485 children were swabbed and 519 *S. aureus* strains were isolated. The second carriage study was also performed on outpatients attending AHC in 2012; the anterior nares of 1058 were swabbed with 261 *S. aureus* strains isolated (unpublished).

The sample size calculation that we performed when deciding how many control samples to sequence are below:
Minimum effect size detectable with 80% power assuming a significance threshold of 5%

Pyomyositis (n=116 cases) versus

	100 controls	200 controls	400 controls	800 controls
Common causal variant (10% minor allele freq)	2.91	2.49	2.27	2.16
Rare causal variant (1% minor allele freq)	10.53	7.64	6.13	5.35

Given there was little statistical difference between sequencing 400 and 800 controls, we made the decision to sequence 400 controls with approximately half of this number sampled in 2008 and half sampled in 2012. 222 isolates were sampled from 2008 and 195 from the 2012 cohort.
Although 116 samples were thought to be available at the beginning, many of these were duplicate samples and were not included in the sequencing and a few of the samples also failed sequencing.

Replicates

- You should report how often each experiment was performed
- You should include a definition of biological versus technical replication
- The data obtained should be provided and sufficient information should be provided to indicate the number of independent biological and/or technical replicates
- If you encountered any outliers, you should describe how these were handled
- Criteria for exclusion/inclusion of data should be clearly stated
- High-throughput sequence data should be uploaded before submission, with a private link for reviewers provided (these are available from both GEO and ArrayExpress)

Please outline where this information can be found within the submission (e.g., sections or figure legends), or explain why this information doesn't apply to your submission:

Plate plans were prepared for both quality assurance and quality control. The cases and controls were randomly assigned, and plates and were mixed up with cases and controls randomly assigned wells in the plates. Each plate contained a previously sequenced *S. aureus* isolate (MRSA252) together with 5 interplate, intrasample repeats. These repeats were randomly assigned wells in the plate. The sequences were all compared during the analysis process.

Quality control of sequencing reads is integrated into the bioinformatics pipeline (and details are provided in cited references). Of short read sequences that passed the sequencing pipeline, at least 83% of reads mapped to the pipeline standard reference genome (MRSA252), with a minimum reference coverage of 83.5%.

All repeat pairs that passed quality checks in read processing were examined: Of 31 duplicate pairs, 29 showed 0 SNPs on mapping calls, 1 pair showed a single base difference and 1 pair showed 3 differences, indicating a very high level of reproducibility in sequencing results.

eLife Sciences Publications, Ltd is a limited liability non-profit non-stock corporation incorporated in the State of Delaware, USA, with company number 5030732, and is registered in the UK with company number FC030576 and branch number BR015634 at the address 1st Floor, 24 Hills Road, Cambridge CB2 1JP | August 2014

Statistical reporting

- Statistical analysis methods should be described and justified
- Raw data should be presented in figures whenever informative to do so (typically when N per group is less than 10)
- For each experiment, you should identify the statistical tests used, exact values of N, definitions of center, methods of multiple test correction, and dispersion and precision measures (e.g., mean, median, SD, SEM, confidence intervals; and, for the major substantive results, a measure of effect size (e.g., Pearson's r, Cohen's d)
- Report exact p-values wherever possible alongside the summary statistics and 95% confidence intervals. These should be reported for all key questions and not only when the p-value is less than 0.05.

Please outline where this information can be found within the submission (e.g., sections or figure legends), or explain why this information doesn't apply to your submission:

Figure 2;
Materials and Methods: Variant calling; Genome wide association testing of Kmers; Testing for lineage effects; Multiple testing correction.

(For large datasets, or papers with a very large number of statistical tests, you may upload a single table file with tests, Ns, etc., with reference to sections in the manuscript.)

Group allocation

- Indicate how samples were allocated into experimental groups (in the case of clinical studies, please specify allocation to treatment method); if randomization was used, please also state if restricted randomization was applied
- Indicate if masking was used during group allocation, data collection and/or data analysis

Please outline where this information can be found within the submission (e.g., sections or figure legends), or explain why this information doesn't apply to your submission:

Case and control allocation is discussed in methods (Patient sample collection). To avoid batch effects in sequencing, isolates from cases and controls were randomly mixed using the excel randomiser and interspersed throughout the sequencing plates.

Additional data files ("source data")

- We encourage you to upload relevant additional data files, such as numerical data that are represented as a graph in a figure, or as a summary table
- Where provided, these should be in the most useful format, and they can be uploaded as "Source data" files linked to a main figure or table
- Include model definition files including the full list of parameters used
- Include code used for data analysis (e.g., R, MatLab)
- Avoid stating that data files are "available upon request"

Please indicate the figures or tables for which source data files have been provided:

Data availability. Sequence data has been submitted to SRA (Bioproject ID PRJNA418899). All of the R code used in this project are already publically available.

Reference

Nickerson, E. K., V. Wuthiekanun, V. Kumar, P. Amornchai, N. Wongdeethai, K. Chheng, N. Chantratita, H. Putchhat, J. Thaipadungpanit, N. P. Day and S. J. Peacock (2011). "Emergence of community-associated methicillin-resistant Staphylococcus aureus carriage in children in Cambodia." The American Journal of Tropical Medicine and Hygiene **84**(2): 313-317.
